# Transmission dynamics and control of two epidemic waves of SARS-CoV-2 in South Korea

**DOI:** 10.1186/s12879-021-06204-6

**Published:** 2021-05-26

**Authors:** Sukhyun Ryu, Sheikh Taslim Ali, Eunbi Noh, Dasom Kim, Eric H. Y. Lau, Benjamin J. Cowling

**Affiliations:** 1 Department of Preventive Medicine, Konyang University College of Medicine, Daejeon, 35365 Republic of Korea; 2 WHO Collaborating Centre for Infectious Disease Epidemiology and Control, School of Public Health, Li Ka Shing Faculty of Medicine, The University of Hong Kong, Hong Kong, Special Administrative Region China; 3 Laboratory of Data Discovery for Health, Hong Kong Science and Technology Park, New Territories, Hong Kong Special Administrative Region China; 4 Graduate School of Public Health, Seoul National University, Seoul, 08826 Republic of Korea

**Keywords:** COVID-19, SARS-CoV-2, Transmission, Epidemiology, Social distancing measure

## Abstract

**Background:**

After relaxing social distancing measures, South Korea experienced a resurgent second epidemic wave of coronavirus disease 2019 (COVID-19). In this study, we aimed to identify the transmission dynamics of severe acute respiratory syndrome coronavirus 2 (SARS-CoV-2) infections and assess the impact of COVID-19 case finding and contact tracing in each epidemic wave.

**Methods:**

We collected data on COVID-19 cases published by local public health authorities in South Korea and divided the study into two epidemic periods (19 January–19 April 2020 for the first epidemic wave and 20 April–11 August 2020 for the second epidemic wave). To identify changes in the transmissibility of SARS-CoV-2, the daily effective reproductive number (*R*_*t*_) was estimated using the illness onset of the cases. Furthermore, to identify the characteristics of each epidemic wave, frequencies of cluster types were measured, and age-specific transmission probability matrices and serial intervals were estimated. The proportion of asymptomatic cases and cases with unknown sources of infection were also estimated to assess the changes of infections identified as cases in each wave.

**Results:**

In early May 2020, within 2-weeks of a relaxation in strict social distancing measures, *R*_*t*_ increased rapidly from 0.2 to 1.8 within a week and was around 1 until early July 2020. In both epidemic waves, the most frequent cluster types were religious-related activities and transmissions among the same age were more common. Furthermore, children were rarely infectors or infectees, and the mean serial intervals were similar (~ 3 days) in both waves. The proportion of asymptomatic cases at presentation increased from 22% (in the first wave) to 27% (in the second wave), while the cases with unknown sources of infection were similar in both waves (22 and 24%, respectively).

**Conclusions:**

Our study shows that relaxing social distancing measures was associated with increased SARS-CoV-2 transmission despite rigorous case findings in South Korea. Along with social distancing measures, the enhanced contact tracing including asymptomatic cases could be an efficient approach to control further epidemic waves.

**Supplementary Information:**

The online version contains supplementary material available at 10.1186/s12879-021-06204-6.

## Background

Coronavirus disease 2019 (COVID-19) is an infectious disease caused by severe acute respiratory syndrome coronavirus 2 (SARS-CoV-2) infection [1]. It was first reported in December 2019 in Wuhan, China [2], and the World Health Organization declared it a public health emergency of international concern on 30 January 2020 [3]. South Korea became the third country to report an imported COVID-19 case on 19 January 2020 [4]. Further spread of COVID-19 in the community was reported in mid-February, and the Korean Ministry of Health and Welfare declared the highest level of public health alert on 23 February 2020 [5]. Since the first COVID-19 case was identified in South Korea, isolation of confirmed cases, contact tracing, extensive testing, and timely quarantine of all contacts have been conducted under the strategic guidelines for COVID-19 control from Korea Centers for Disease Control and Prevention (KCDC) [6, 7]. Furthermore, combined public health measures including travel-related measures, case-based measures, and community measures were implemented across South Korea which helped control the first epidemic wave without a complete lockdown (Table S1) [5, 8]. Between 22 March and 19 April 2020, strict social distancing measures included recommendations to the public to stay at home and to delay or cancel social gatherings, as well as policies including closing schools and other public facilities, allowing greater flexibility in sick leave, and encouraging work-from-home and flexible working hours [9, 10]. These strict social distancing measures were relaxed on 20 April 2020, because the daily reported number of cases was under 50 and the unknown origin of infection was less than 5% among total cases of investigation for the previous 2 weeks [11].

Sustained increases in cases were observed as the strict social distancing measures were further relaxed by opening public facilities on 6 May 2020 when the first epidemic wave had already ended. Increase of the cases can be observed by the changes of public health efforts of extensive COVID-19 case finding and contact tracing [12]. However, there is a lack of study to assess the changes of this active case finding strategy specifically.

To characterize the transmission dynamics of SARS-CoV-2 in South Korea, we identified the major cluster types of COVID-19 cases, and we estimated the time-varying effective reproductive number, serial interval distribution, age-specific infector-infectee matrices for the two COVID-19 epidemic waves. Furthermore, to assess public health efforts to find and trace cases in South Korea, we estimated the proportion of cases that were asymptomatic and the proportion of local cases with an unknown origin of infection at the time of first clinical assessment.

## Methods

### Data sources

South Korea is comprised of a special city, seven metropolitan cities and nine provinces, with a total population of 51.4 million. Among the 14,700 confirmed COVID-19 cases in South Korea to date (16 August 2020), 8358 (57%) cases were reported from the Daegu-Gyeongsanbuk region (5.1 million population), including 5564 cases from a large initial cluster linked to a religious community [13, 14].

We collected data on COVID-19 cases that had been confirmed by real-time reverse transcriptase-polymerase chain reaction (RT-PCR) in South Korea. Nasal swab samples from all suspected contacts of COVID-19 confirmed cases were tested at the KCDC or national designated laboratories, following a consistent test protocol [15]. Data in the early COVID-19 epidemics from the Daegu-Gyeongsanbuk region have not been made publicly available [16].

Local public health authorities provided daily updates of all laboratory-confirmed COVID-19 cases on their webpage and we compiled daily updates from the public dashboard in different local authorities. Information included national and local case number, age, sex, symptoms, symptom onset date, source, exposure date, and location (within or outside South Korea) of infection were provided. We also compiled a database of COVID-19 infector-infectee transmission pairs where precise symptom onset dates and source of infection were available. Here, we focused on the period from 19 January through 11 August 2020, after which the specific information of cases was not yet released by the local authorities under the reinforced regulation of privacy protection [17].

### Definitions

Based on the epidemic curve and the timing of relaxation of social distancing measures, we defined the first epidemic wave of COVID-19 as the period between 19 January 2020–19 April 2020, and the second epidemic wave between 20 April – 11 August 2020. Furthermore, we defined community clusters as five or more linked COVID-19 cases [18], excluding cases with epidemiologic links to the secondary transmission and within household transmissions [18, 19]. The types of clusters included musical events, religious activities, leisure activities, nosocomial infections, residential homes for the elderly, shopping malls, workplaces, academic-related, and restaurants [19]. The workplace includes logistic facilities, call centres, insurance companies, military facilities, banks, governmental offices, and sales offices in this study. We used the officially agreed cluster settings, and we matched the cases in the cluster reported in each region using the national number of cases. Unlinked local cases were those in which the source of infection has not been found. For the transmission pairs, we defined the infector as the primary COVID-19 case, which was identified from the source of infection from the case, and infectee is the secondary case from the infector.

### Statistical analysis

We compared the differences in age groups between two different waves using chi-squared tests. We described the epidemiological characteristics of COVID-19 cases and the epidemic curve was constructed by stratifying the data into local and imported cases. To identify the potential changes in SARS-CoV-2 transmissibility of local cases after relaxing social distancing measures, we estimated the time-varying effective reproductive number (*R*_*t*_), which defines the mean number of secondary infectious cases generated from a typical primary infectious case at time *t* [20]. We also included local clustered cases to estimate *R*_*t*_. We estimated *R*_*t*_ using the *EpiEstim* package in R [20, 21]. The critical threshold of *R*_*t*_ is 1, where the epidemic becomes under control if *R*_*t*_ falls below this threshold sustainably. *R*_*t*_ was estimated based on the respective serial interval distributions, estimated from the data for the first and second epidemic waves in this study and the daily incidence of COVID-19 cases over time with a 7-day smoothing window [22]. We presented daily *R*_*t*_ after 1 February 2020, because a stable estimate of *R*_*t*_ was not available due to the low number of cases. We computed the serial interval, which represents the time between a symptom onset of successive cases in a transmission chain, as the number of days between infector and that of infectee in the transmission pairs. We estimated the serial interval distribution during the first and second epidemic waves by fitting a normal distribution [5, 22, 23]. We also presented the number of clusters by categories and the frequencies for respective age-groups of infector and infectee using age-specific infector-infectee matrices in both epidemic waves. Furthermore, to identify the changes of comprehensive COVID-19 case finding and contact tracing, the proportions of asymptomatic cases at presentation and cases with an unknown origin of infection among the local cases were calculated, with 95% confidence intervals estimated by the exact binomial method [24]. Analyses were done in R version 3.6.1 (R Foundation for Statistical Computing, Vienna, Austria).

## Results

As of 11 August 2020, 5002 confirmed cases (2038 and 2964 cases in the first and second wave, respectively), including 1189 imported cases (547 and 642 cases in the first and second wave), have been reported outside of Daegu-Gyeongsanbuk region in South Korea. There were no statistically significant differences in age groups between the cases from the two epidemic waves (*p*-value = 0.22; Table S2).

### Transmission dynamic of SARS-CoV-2 in South Korea

**Fig. 1:**
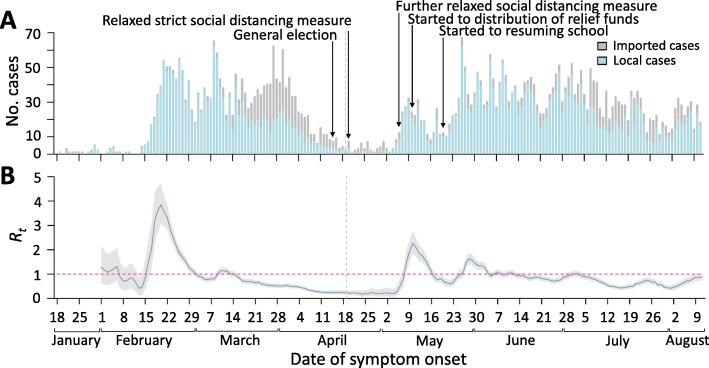
Incidence of COVID-19 and transmissibility of SARS-CoV-2 in South Korea. **A** The reported number of confirmed COVID-19 cases by symptom onset date outside of the Daegu-Gyeongsangbuk region in South Korea. The dates of first clinical assessment were used for cases who were asymptomatic at presentation. There were key events against the spread of SARS-CoV-2 including a general election (on 15 April 2020), relaxing strict social distancing measures (from 20 April 2020) and relaxing the social distancing measure further (from 6 May 2020), distribution of COVID-19 relief funds to the general public (from 11 May 2020) and resuming school (from 20 May 2020). **B** Estimated daily *R*_*t*_ of SARS-CoV-2 in blue line with 95% credible interval in light grey shaded area. The red horizontal dashed line indicates the critical threshold of *R*_*t*_ = 1. The vertical grey dashed line divided the study period on first epidemic waves (19 January–19 April 2020) and second epidemic waves (20 April–11 August 2020). Notes: COVID-19 = coronavirus disease 2019, SARS-CoV-2 = severe acute respiratory syndrome coronavirus 2, *R*_*t*_ = effective reproductive number

Figure 1 shows the epidemic curve with the event timeline and the corresponding daily estimates of *R*_*t*_ between 19 January and 11 August 2020. In early February, after the implementation of combined public health measures, *R*_*t*_ gradually decreased to below the critical threshold of 1 March. After strict social distancing measures were implemented on 22 March, *R*_*t*_ further reduced to below 0.2 in April. On 8 May, 3 days after the strict social distancing measures were further relaxed on 6 May, *R*_*t*_ increased above 1 and eventually reached 1.8 on 10 May. *R*_*t*_ fell to 1 in late May and fluctuated around 1 until early July.

**Fig. 2:**
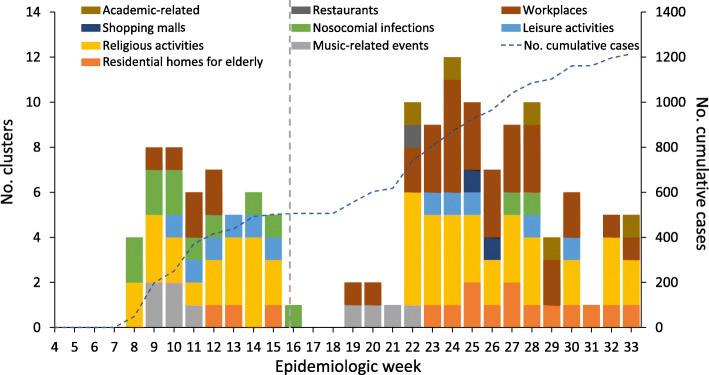
Types of clusters in South Korea in the first and second epidemic waves. Temporal distribution of the clusters of COVID-19 cases outside of the Daegu-Gyeongsangbuk region in South Korea. The vertical grey dashed line divided the study period on first epidemic wave (19 January–19 April 2020) and second epidemic wave (20 April–11 August 2020)

We identified 65 clusters, including religious activities (*n* = 24, 36.9% of total clusters), workplaces (17, 26.1%), institutional homes for the elderly (7, 10.7%), and a number of other settings (Fig. 2 and Fig. S2). The overall number of cases in these clusters was 1212 and the median number of cases in a cluster was 44 (maximum 190). Religious activities were the most frequently reported cluster type in both waves and the number of workplace clusters was largely increased from 6 (first wave) to 30 (second wave).

**Fig. 3:**
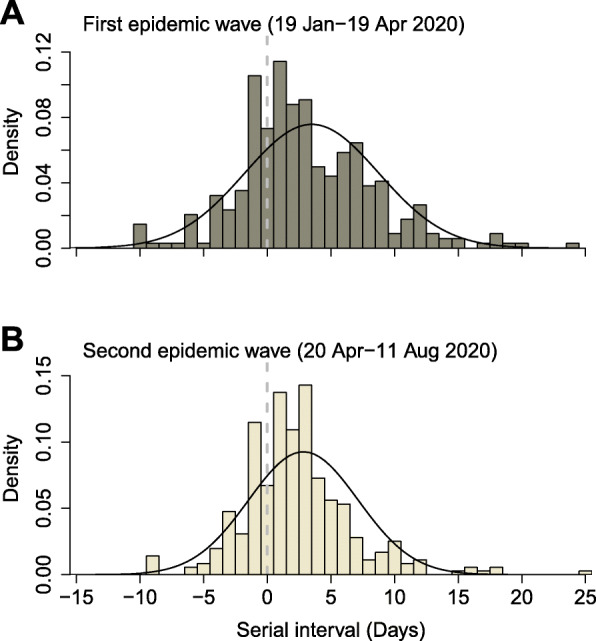
Serial interval distribution of SARS-CoV-2 in the first and second epidemic waves in South Korea. The estimated serial interval distribution was analysed by using the 708 infector-infectee pairs. The vertical bars indicate the empirical probability density of serial interval calculated by constructing transmission pairs from illness onset of confirmed cases and black lines indicate fitted normal distribution (accounting for the possible negative serial intervals/pre-symptomatic transmissions and symmetric pattern of empirical density). Infector who reported symptoms onset in the first epidemic wave (19 January–19 April 2020; 345 pairs) (**A**), and second pandemic wave (20 April–11 August 2020; 363 pairs) (**B**). The left of vertical dashed line in grey indicates definite pre-symptomatic transmission

**Fig. 4:**
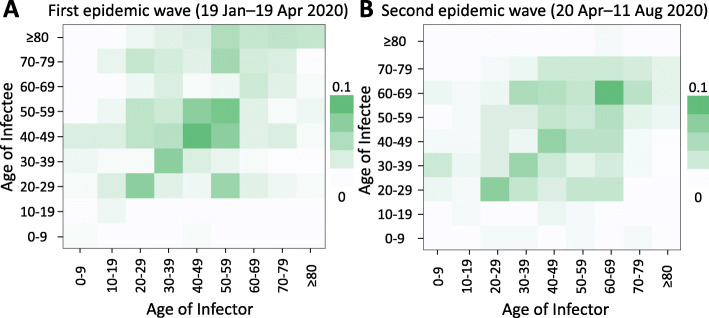
Age-specific transmission matrix in South Korea **A** Infector-infectee matrix for the first epidemic wave (19 January–19 April 2020). The colour in each cell represents the probability of infector-infectee pairs of the respective ages. **B** Infector-infectee matrix for the second epidemic wave (20 April–11 August 2020)

We identified 707 transmission pairs, including 345 in the first wave and 362 in the second wave. We excluded 4 pairs in the first wave and 5 pairs in the second wave which had large negative serial intervals (<− 10 days) [25]. In the first epidemic wave, the mean and standard deviation of serial intervals were estimated to be 4.0 days (95% credible interval, CrI: 3.7, 4.3) and 5.3 days (95% CrI: 5.1, 5.5), respectively (Fig. 3A). In the second epidemic wave, the mean and standard deviation were 3.2 days (95% CrI: 3.0, 3.5) and 4.5 days (95% CrI: 4.3, 4.6), respectively (Fig. 3B). Around 90% of transmission took place among the age-groups of 20–80 years with ~ 30% intra age-group transmissions in both waves (Fig. 4; Tables S3, S4). Compared to the first wave, transmissions among the 40–59 age groups were less prominent in the second wave and the oldest age group (≥80 years) were much less affected. On the other hand, transmission in the second wave increased for the infectees of age-groups 30–39 and 60–69 by 7 and 18%, respectively.

### Assessment of active case finding

**Fig. 5:**
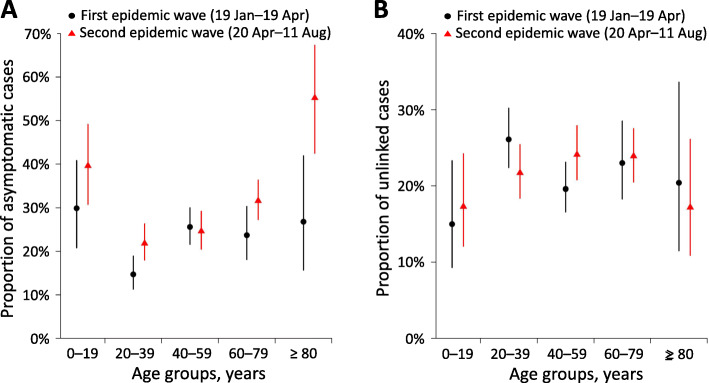
The proportions of asymptomatic and unlinked local cases of COVID-19 in South Korea. **A** Age-specific proportions of COVID-19 cases who were asymptomatic at the time of presentation in South Korea. **B** Age-specific proportion of infections of an unknown origin in South Korea. The points were average proportions over the epidemic wave and the vertical bars indicate 95% confidence intervals estimated by the binomial method. Notes: COVID-19 = coronavirus disease 2019

Overall, 634 cases (16.6% of all local cases) reported no symptoms at the time of the first clinical assessment. The mean proportion of asymptomatic cases at presentation in those aged 20–39 years increased from 14.7 to 21.8% over time, and the overall mean proportion increased from 21.7 to 27.1% (Fig. 5A; Table S5). There were a total of 758 unlinked cases in the study period. The overall mean proportion of unlinked cases was similar across the two epidemic waves (22.0 and 22.5%) (Fig. 5B; Table S6).

## Discussion

In South Korea, the combined public health measures implemented early in the COVID-19 epidemic reduced the spread of SARS-CoV-2 [5]. As mobility restriction has led to a substantial economic and social loss [26], many countries including South Korea relaxed social distancing measures after the first epidemic wave despite concerns of a resurgence of COVID-19 cases. Our analysis of two different epidemic waves provided insight into the strategies to control COVID-19, particularly in countries that have relaxed their social distancing measures.

A large number of imported cases in the first epidemic wave challenged efforts to control COVID-19 in South Korea. In late March 2020, many Koreans in Europe and the United States traveled back to Korea due to the lockdowns. Public health authorities implemented mandatory laboratory screening at the port of entry and a 14-day mandatory self-quarantine program for travellers from Europe (22 March) and then all overseas travellers (1 April) [27]. Local health authorities identified 547 imported cases and they were linked with 44 local cases during the first wave (Fig. S1). Furthermore, there were 642 imported cases linked with 3 local cases (most from Asia and North America) identified during the second wave. To encourage the 14-day mandatory self-quarantine for travellers arriving in South Korea, the Korean government implemented strategies to support (by providing financial aid), monitor (by using mobile phone application), and sanction (by enhancing noncompliance penalty and subjecting to deportation) the individuals in self-quarantine [27]. Therefore, the control of transmission from imported cases improved substantially in the second wave and the re-emergence was mainly driven by local transmission in the community.

We identified many clusters associated with religious activities where individuals spend prolonged periods of time in close proximity [14]. These were the most frequent type of clusters reported in both first and second waves. Although the Korean government emphasized wearing face masks and keeping physical distance between persons during their meeting, transmission still occurred, possibly through the droplet-borne route or environmental contamination of shared surfaces [28]. Resuming economic activities and school early in the second wave (COVID-19 relief funds started distribution to the public on 11 May and resuming school on 20 May) was likely associated with clustered outbreaks in workplace, shopping malls, and academic institutions, and this has likely contributed to the longer duration of *R*_*t*_ around 1 more than a month.

We found that a large proportion of transmission occurred between individuals of the same age, and transmission between children was rare. However, school closure in the first wave may have contributed to reduced opportunities for transmissibility between children [29]. After resuming school, the active daily-screening, monitoring, and following personal protective measures to the students may also reduce the risk of transmission between children in the second wave.

We identified that 16.6% of the local cases were asymptomatic at the time of presentation, which is similar to those in Shenzhen (20%) and Hong Kong (21%) in China [30, 31]. The proportion of asymptomatic cases was lower in the first epidemic wave, particularly among those aged 20–39 years. This is likely because the case investigations were mainly symptom-based and required an epidemiological link in the earlier period. The emerging scientific evidence on the full spectrum of SARS-CoV-2 infections encouraged more testing on asymptomatic individuals. Young adults may have lower or delayed healthcare-seeking [32], which may explain their lowest proportion of asymptomatic cases among all age groups. In the second epidemic wave, extensive contact tracing and screening for the large workplace and leisure-related clusters, probably allowed the detection of infected young adults who were still asymptomatic or pre-symptomatic. This suggests that active case finding and improving the awareness of the disease dynamics among young adults is crucial to reducing asymptomatic and pre-symptomatic transmission of SARS-CoV-2. The proportion of asymptomatic cases among 80s increased significantly from the first to second epidemic wave. This was likely affected due to rapid and massive screening of the elderly for SARS-CoV-2 in residential homes during the second epidemic waves.

In South Korea, all close contacts of laboratory-confirmed cases were quarantined, and public health authorities diligently traced the source of infection. The presence of unidentified cases indicates hidden and uncontrolled transmission in the community. Therefore, a low proportion of unlinked local cases is an indication of effective case finding [33]. In our study, 20% of local cases were identified as unlinked, which was lower than the early phase of the epidemic (39%, 18 January – 2 March 2020) [34], similar to that in Singapore (17%) [33], but lower than that in Hong Kong (36%) [35]. The proportions of unlinked local cases were low in the two epidemic waves and this indicates that extensive investigation was maintained in both waves.

A study in China demonstrated that serial interval distributions can be shortened by active case finding and enhanced public health measures [22]. In our study, the mean serial interval was about 3 days, shorter than those reported in China (4 days) [23], and a pooled estimate of 5 days [36]. The implementation of rigorous public health measures, including registered mandatory digital applications (QR codes) in public places for contact tracing may contribute to earlier interrupt of the transmission of SARS-CoV-2. A negative number of serial intervals indicates the symptom onset in the infectee occurs prior to symptom onset in the infector. Based on the previous literature on the serial interval of COVID-19 [25], large negative serial intervals in some of transmission pairs in our study should be interpreted with caution as there could be uncertainty of the direction of transmission or the infectee could have been infected by another unidentified infector.

A modelling study demonstrated that control measures, including contact tracing, testing, and self-isolation, would be less effective if asymptomatic infections are higher [37]. Furthermore, a review study showed that restricting mass gathering was associated with a reduced incidence of COVID-19 [38]. Our findings are consistent with these previous findings that continuous, strict social distancing measures and active seeking asymptomatic cases are critical to reducing the spread of SARS-CoV-2 in a community.

Recent studies in Hong Kong, China [39], and South Korea [5] demonstrated that social distancing measures were effective in controlling COVID-19. However, there have been few studies on the control of resurgences in transmission after the relaxation of social distancing measures. A simulation study also demonstrated that the second wave of infection would develop when contact tracing failed [40]. Our findings of *R*_*t*_ indicated that even when rigorous public health efforts were in place, relaxation of certain social distancing measures in the community, in our case further reopening of public facilities, may allow resurgence of COVID-19 within days. Further research on how social distancing and other public health measures should be relaxed is warranted. Furthermore, simulation studies based on empirical data with the counterfactual scenarios to predict a potential resurgence of COVID-19 would help public health authorities prepare for future outbreaks.

Our analysis has several limitations. First, we excluded the Daegu-Gyeongsanbuk region where the epidemic was mainly driven by large superspreading events in a religious group at the very beginning of the first epidemic wave. The outbreak occurred mainly in Daegu-Gyeongsanbuk region and well before the major control measures were implemented, and uncooperative attitude of the members of the religious group was reported during the epidemiological investigation [14]; hence it did not reflect characteristics of typical community transmission in South Korea. Furthermore, the Korean government designated the Daegu-Gyeongsanbuk region as a special disaster zone and recommended travel with caution during the study period [41]. Second, we have not included data after mid-August 2020 which reported a number of clusters from religious groups as publicly available data were limited. Third, recall bias could affect the description of symptom onset and the exposure period of the infectee. Fourth, asymptomatic cases who were not identified and the imperfect sensitivity of the RT-PCR test may affect the estimated transmissibility in our study. Fifth, local public health authorities may identify the infection source after publishing the case-information, which may overestimate the proportion of unlinked local cases. Sixth, due to data limitation, we have not considered the spatial transmission heterogeneity along with the temporal variations in this study. Lastly, the effects of seasonality on SARS-CoV-2 were not considered which may partly explain the change in transmissibility [42].

Our study has several strengths including the estimation of daily *R*_*t*_ of SARS-CoV-2 in South Korea from illness onset data, whereas previous studies [6, 43, 44] estimated it from the daily confirmed cases, which might be subject to reporting bias [45]. Furthermore, we estimated *R*_*t*_ using the serial interval distributions, which is evaluated by constructing transmission pairs on this illness onset data. Whereas, the earlier estimates were based on the approximation of serial interval distributions, evaluated for different data and locations, mostly used the early finding of serial interval in China which did not even include the pre-symptomatic transmission [46] and might have been different over time (here for two epidemics) [22]. Third, we included all local clustered cases to estimate *R*_*t*_ to better characterize the changes of transmissibility after relaxing social distancing measures which did not include earlier study [5]. Finally, our study has an added value over our previous report [5], providing a more detailed interpretation of the transmission dynamics by accounting for local clustered cases [36], and included changes in transmissibility after relaxing social distancing measures in South Korea.

## Conclusions

South Korea has implemented public health measures to reduce the transmissibility of SARS-CoV-2. However, despite rigorous public health efforts, relaxing social distancing measures allowed the reemergence of COVID-19 in South Korea. This study is the first epidemic analysis of the two different epidemic waves of COVID-19 using empirical data based on the illness onset and serial interval which analysed in our transmission pairs. Our findings suggest that enhanced contact tracing including asymptomatic cases is an efficient approach to control further epidemic waves together with social distancing. A more conservative approach to control COVID-19 should be considered together with close monitoring of social mobility.

## Supplementary Information

**Additional file 1.**

## Data Availability

The datasets used in the current study are available at https://github.com/gentryu/Korean_two_wave_COVID19.
