# Correction to: Transmission dynamics and control of two epidemic waves of SARS-CoV-2 in South Korea

**DOI:** 10.1186/s12879-021-06358-3

**Published:** 2021-07-07

**Authors:** Sukhyun Ryu, Sheikh Taslim Ali, Eunbi Noh, Dasom Kim, Eric H. Y. Lau, Benjamin J. Cowling

**Affiliations:** 1 Department of Preventive Medicine, Konyang University College of Medicine, Daejeon, 35365 Republic of Korea; 2 WHO Collaborating Centre for Infectious Disease Epidemiology and Control, Li Ka Shing Faculty of Medicine, School of Public Health, The University of Hong Kong, Hong Kong, Hong Kong, Special Administrative Region of China; 3 Laboratory of Data Discovery for Health, Hong Kong Science and Technology Park, New Territories, Hong Kong, Special Administrative Region of China; 4 Graduate School of Public Health, Seoul National University, Seoul, 08826 Republic of Korea

**Correction to: BMC Infect Dis. 21, 485 (2021)**

**https://doi.org/10.1186/s12879-021-06204-6**

Following publication of the original article [1], the authors identified errors in the abstract, article body, Fig. 1. Fig 5. and Table S6.

- The typo “24%” in the 1st page, 3rd paragraph, should be written as ‘25%’.
- The typo “547 and 642 cases” in the 3rd page, left-hand column, 3rd paragraph, should be written as ‘555 and 634’.
- The typo “65 clusters”,“36.9%”, “26.1%”, and “10.7%” in the 3rd page, right-hand column, 3rd paragraph, should be written as ‘67 clusters’, ‘35.8%’, ‘25.4%’, and ‘10.5%’, respectively.
- The typo “758” in the 3rd page, right-hand column, 5th paragraph, and “22.5%” in the 4th page, left-hand column, 1st paragraph should be written as ‘916’, and ‘25.3%’.
- The typo “708 infector-infectee pairs”,“345 pairs”, and “363 pairs” in the Fig. 3. legend, in the 5th page, left-hand column, should be written as ‘698 infector-infectee pairs’, ‘341 pairs’, and ‘357 pairs’, respectively.
- The typo “547 imported cases” and “642 imported cases” in the 5th page, right-hand column, 1st paragraph, should be written as ‘555 imported cases’ and ‘634 imported cases’, respectively.
- The corrected Fig. 1 is given below.

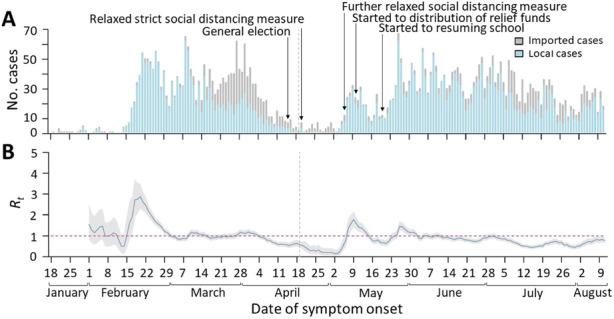

- The corrected Fig. 5 is given below

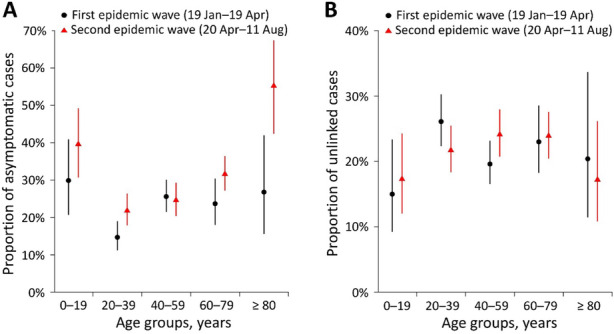

- The corrected Table S6 is given below.

**Table:** 

	First epidemic wave			Second epidemic wave		
Age group (years)	No. of unlinked cases	No. of confirmed cases	Proportion of unlinked (95% CI^†^), %	No. of unlinked cases	No. of confirmed cases	Proportion of unlinked (95% CI^†^), %
0–19	15	100	15.0 (9.3–23.3)	26	150	17.3 (12.1–24.2)
20–39	124	475	26.1 (22.4–30.2)	117	539	21.7 (18.4–25.4)
40–59	113	576	19.6 (16.6–23.1)	133	551	24.1 (20.8–27.9)
60–79	60	261	23.0 (18.3–28.5)	136	570	23.9 (20.5–27.5)
≥80	10	49	20.4 (11.5–33.6)	16	93	17.2 (10.9–26.1)
NA	4	22	18.2 (7.3–38.5)	162	427	37.9 (33.5–42.6)
Overall	326	1483	22.0 (19.9–24.2)	590	2330	25.3 (23.6–27.1)
