# Supplementary material for: Transmission dynamics and control of two epidemic waves of SARS-CoV-2 in South Korea
Source: BMC Infect Dis. 2021 May 26;21:485. doi: 10.1186/s12879-021-06204-6 (PMC8154110; doi:10.1186/s12879-021-06204-6)
Supplement: Supplementary file 1 — Additional file 1. [file 12879_2021_6204_MOESM1_ESM.docx]

**Supplementary file**

**Title:** Transmission dynamics and control of two epidemic waves of SARS-CoV-2 in South Korea

**Authors:**

Sukhyun Ryu^1^, Sheikh Taslim Ali^2,3^, Eunbi Noh^1,4^, Dasom Kim^1^, Eric H.Y. Lau^2,3^, Benjamin J. Cowling^2,3^

**Affiliations:**

1. Department of Preventive Medicine, Konyang University College of Medicine, Daejeon, 35365, Republic of Korea

2. WHO Collaborating Centre for Infectious Disease Epidemiology and Control, School of Public Health, Li Ka Shing Faculty of Medicine, The University of Hong Kong, Hong Kong Special Administrative Region, China

3. Laboratory of Data Discovery for Health, Hong Kong Science and Technology Park, New Territories, Hong Kong Special Administrative Region, China.

4. Graduate School of Public Health, Seoul National University, Seoul, 08826, Republic of Korea

**Corresponding author:** Prof Sukhyun Ryu, Department of Preventive Medicine, Konyang University College of Medicine, R707, Myungok-Euihak Gwan, 158, Gwanjeodong-ro, Seogu, Daejeon, 35365, South Korea. Telephone: +82 42 600 8673; Email: gentryu@onehealth.or.kr

# Contents:

1. Supplementary Figures
2. Supplementary Tables

# 1. Supplementary Figures

**Figure S1. Imported cases of SARS-CoV-2 in South Korea by country of infection.** The vertical dashed line divided the study period on the first epidemic waves (19 January–19 April, 2020) and the second epidemic period (20 April–11 August, 2020).


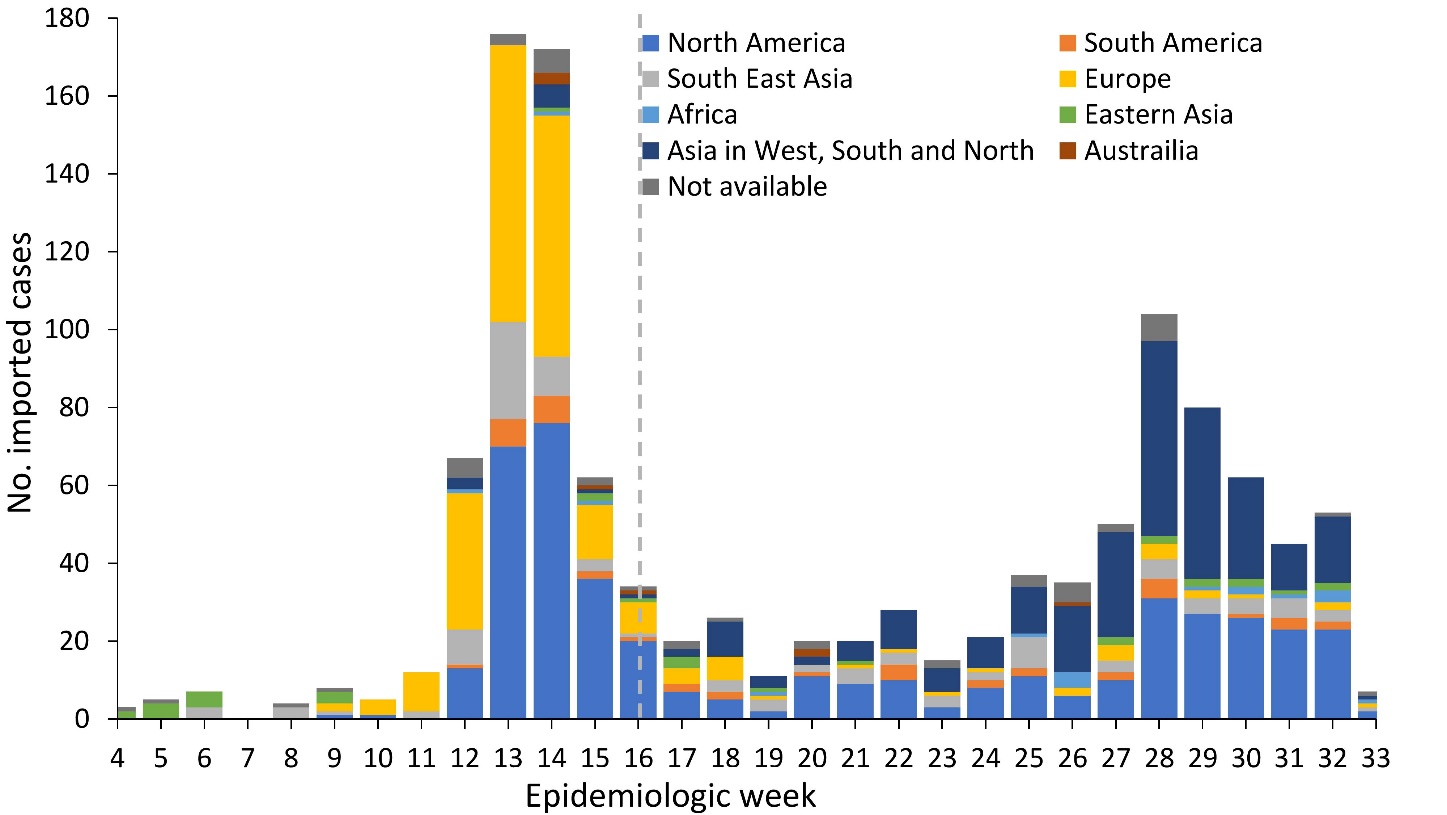


**Figure S2. Probability distribution of the number of sizes by cluster type in South Korea.** Probability density distribution of the number of COVID-19 cases in each cluster type including (A) music-related events, (B) workplaces, (C) religious activities, (D) nosocomial infection, (E) residential home for elderly, (F) leisure activities, (G) academic-related, and shopping mall.

**
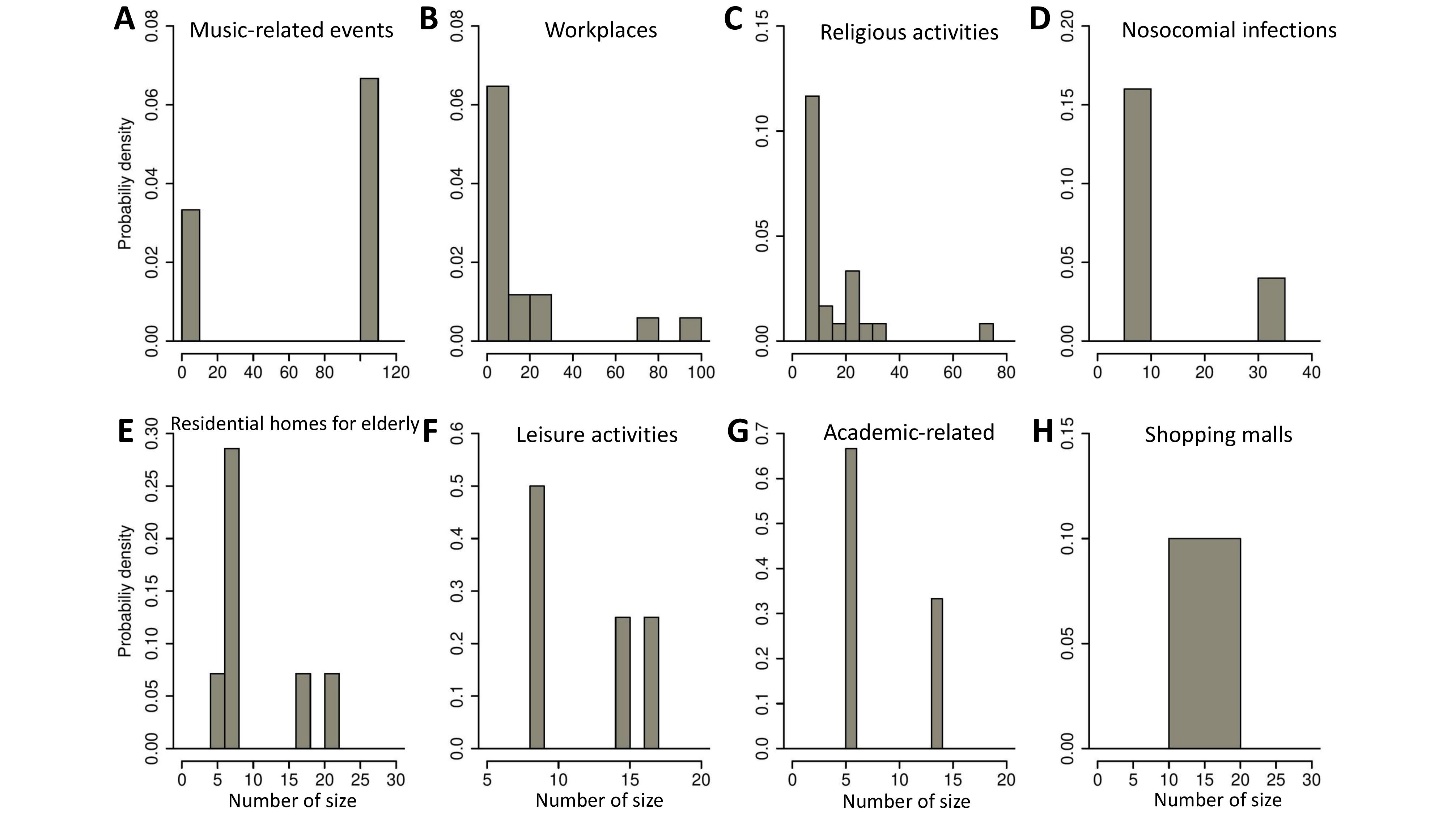
**

# 2. Supplementary Tables

Table S1. Non-pharmaceutical interventions in South Korea (most of the key interventions)

| **Starting date** | **Interventions** | **Source** |
| --- | --- | --- |
| **Travel related measures** | | |
| February 3, 2020 | Barred of entry of foreign travelers from Hubei province, China | http://overseas.mofa.go.kr/ru-ko/brd/m_7329/view.do?seq=1345588&srchFr=&amp;srchTo=&amp;srchWord=&amp;srchTp=&amp;multi_itm_seq=0&amp;itm_seq_1=0&amp;itm_seq_2=0 |
| February 23, 2020 | Recommended travel restriction in Daegu City - Recommended avoiding social gathering and refraining from going out in Daegu city | http://ncov.mohw.go.kr/tcmBoardView.do?contSeq=353064 |
| March 9, 2020 | Barred of entry of foreign travelers from Japan | http://overseas.mofa.go.kr/jp-ko/brd/m_1083/view.do?seq=1343492 |
| April 1, 2020 | Implemented 14-day mandatory quarantine for arriving Korea | http://ncov.mohw.go.kr/tcmBoardView.do?brdId=&brdGubun=&dataGubun=&ncvContSeq=353800&contSeq=353800&board_id=&gubun=ALL |
| July 13, 2020 | Arrivals from 'high-risk nations' required to submit proof of 'negative' virus test results. | ncov.mohw.go.kr/tcmBoardView.do?brdId=&brdGubun=&dataGubun=&ncvContSeq=355351&contSeq=355351&board_id=&gubun=ALL |
| **Case-based measures** | | |
| February 17, 2020 | Implemented screening test for COVID-19 for the health care workers at all nursing home | https://www.cdc.go.kr/board/board.es?mid=a30402000000&bid=0030&act=view&list_no=366586&tag=&nPage=1 |
| February 23, 2020 | Lauched nationwide drive-through screening centers - Operated 58 roadside screening sites to timely identify the infected cases in the community as of May 11, 2020. | https://jkms.org/DOIx.php?id=10.3346/jkms.2020.35.e123  https://www.mohw.go.kr/react/popup_200128_4.html |
| February 25, 2020 | Initiated screening all Sincheonji religious group members - As the Sincheonji religious group occupied a large portion of COVID-19 cases in Korea, Korean public health authorities initiated the screening program for this group members (ca. 0.2 million) | http://www.korea.kr/news/pressReleaseView.do?newsId=156377318 |
| February 27, 2020 | Designated private hospitals as public relief hospital in nationwide - Operated 339 public relief hospitals where COVID-19 screening test is available as of May 11, 2020 | http://ncov.mohw.go.kr/tcmBoardView.do?brdId=&brdGubun=&dataGubun=&ncvContSeq=353184&contSeq=353184&board_id=140&gubun=BDJ  https://www.mohw.go.kr/react/popup_200128_4.html |
| March 9, 2020 | Implemented nationwide screening the elderly at nursing home | https://www.gov.kr/portal/ntnadmNews/2120440 |
| May 11, 2020 | Government offered "anonymous" testing for those visited Itaewon Club who do not want to reveal their identities. | http://ncov.mohw.go.kr/tcmBoardView.do?brdId=&brdGubun=&dataGubun=&ncvContSeq=354535&contSeq=354535&board_id=&gubun=ALL |
| **Community measures** | | |
| February 23, 2020 | Raised the infectious disease alert to the highest level | https://www.cdc.go.kr/board/board.es?mid=a20501000000&bid=0015&act=view&list_no=366324&tag=&nPage=1 |
| February 23, 2020 | Postponed school opening for new semester - School breaks were extended nationwide until the notification by Korean Ministry of Education | https://www.moe.go.kr/boardCnts/view.do?boardID=294&boardSeq=79829&lev=0&searchType=S&statusYN=W&page=1&s=moe&m=020402&opType=N |
| March 9, 2020 | Distributed public face masks - Evenly provided the face masks to the public through the public channels to prevent stockpiling | https://www.mfds.go.kr/brd/m_99/view.do?seq=44020&srchFr=&srchTo=&srchWord=&srchTp=&itm_seq_1=0&itm_seq_2=0&multi_itm_seq=0&company_cd=&company_nm=&page=1 |
| March 22, 2020 | Implemented social distancing measures - Recommended canceling any social event, avoiding social gathering and refraining from going out in nationwide | http://ncov.mohw.go.kr/shBoardView.do?brdId=2&brdGubun=27&ncvContSeq=1385# |
| April 20, 2020 | Softened strict social distancing measures (20 April – 5 May) - Allowed to have the social gathering and travel | http://www.mohw.go.kr/react/al/sal0301vw.jsp?PAR_MENU_ID=04&MENU_ID=0403&page=1&CONT_SEQ=354112 |
| May 5, 2020 | Relaxed social distancing measures | http://ncov.mohw.go.kr/tcmBoardView.do?brdId=&brdGubun=&dataGubun=&ncvContSeq=354377&contSeq=354377&board_id=&gubun=ALL |
| May 20, 2020 | Reopening school (phased plan on May 20 - Jun 8) | https://www.moe.go.kr/boardCnts/view.do?boardID=340&boardSeq=80512&lev=0&m=02  https://www.moe.go.kr/boardCnts/view.do?boardID=294&boardSeq=80575&lev=0&searchType=S&statusYN=W&page=6&s=moe&m=020402&opType=N |
| May 26, 2020 | People without a face mask will be barred from public transport, in a nationwide measure. | http://ncov.mohw.go.kr/tcmBoardView.do?brdId=&brdGubun=&dataGubun=&ncvContSeq=354715&contSeq=354715&board_id=&gubun=ALL |
| Jun 10, 2020 | KI-Pass: Korea Internet - Pass (QR code) scanning becomes mandatory at high-risk facilities. | http://ncov.mohw.go.kr/tcmBoardView.do?brdId=&brdGubun=&dataGubun=&ncvContSeq=354948&contSeq=354948&board_id=&gubun=ALL |
| Jul 10, 2020 | Strengthening prevention measure in Churches | http://ncov.mohw.go.kr/tcmBoardView.do?brdId=3&brdGubun=31&dataGubun=&ncvContSeq=3011&contSeq=3011&board_id=311&gubun=BDC |
| Jul 11, 2020 | End of distributing public face masks | https://terms.naver.com/entry.nhn?docId=5926931&cid=43667&categoryId=43667  http://it.chosun.com/site/data/html_dir/2020/07/07/2020070702349.html |
| August 3, 2020 | All ship crews of countries subject to enhanced quarantine and Russia are required to submit PCR negative certificates. | http://ncov.mohw.go.kr/tcmBoardView.do?brdId=3&brdGubun=31&dataGubun=&ncvContSeq=3260&contSeq=3260&board_id=311&gubun=BDC |
| August 7, 2020 | New quarantine regulations for café | http://ncov.mohw.go.kr/tcmBoardView.do?brdId=3&brdGubun=31&dataGubun=&ncvContSeq=3321&contSeq=3321&board_id=311&gubun=BDC |

**Table S2. Demographic characteristics of COVID-19 cases acquired by local transmission in different epidemic periods outside of the Daegu-Gyeongsangbuk region in South Korea**

|  | First epidemic wave  (19 January–19 April 2020) | | | | Second epidemic wave  (20 April–11 August 2020) | | | |
| --- | --- | --- | --- | --- | --- | --- | --- | --- |
| Age group (years) | Male | Female | NA | All | Male | Female | NA | All |
| 0–19 | 58 (58.0%) | 42 (42.0%) | 0 | 100 | 94 (62.7%) | 53 (35.3%) | 3 (2.0%) | 150 |
| 20–39 | 233 (49.1%) | 239 (50.3%) | 3 (0.6%) | 475 | 319 (59.2%) | 196 (36.4%) | 24 (4.5%) | 539 |
| 40–59 | 227 (39.4%) | 348 (60.4%) | 1 (0.2%) | 576 | 217 (39.4%) | 308 (55.9%) | 26 (4.7%) | 551 |
| 60–79 | 107 (41.0%) | 154 (59.0%) | 0 | 261 | 236 (41.4%) | 296 (51.9%) | 38 (6.7%) | 570 |
| ≥80 | 16 (32.7%) | 33 (67.3%) | 0 | 49 | 28 (30.1%) | 60 (64.5%) | 5 (5.4%) | 93 |
| NA | 3 (13.6%) | 1 (4.5%) | 18 (81.8%) | 22 | 1 (0.2%) | 1 (0.2%) | 425 (99.5%) | 427 |

NA: Not available

**Table S3. Observed age-specific transmission probability matrix in the first epidemic wave in South Korea.** The probability is derived as the density measure by using the respective frequencies for the transmission among the age-groups of infector-infectee.

|  | | Infectee | | | | | | | | | | |
| --- | --- | --- | --- | --- | --- | --- | --- | --- | --- | --- | --- | --- |
|  |  | 0-9 | 10-19 | 20-29 | 30-39 | 40-49 | 50-59 | 60-69 | 70-79 | 80-89 | ≥90 |  |
| Infector | 0-9 | 0.00 | 0.00 | 0.00 | 0.01 | 0.02 | 0.00 | 0.00 | 0.00 | 0.00 | 0.00 |  |
|  | 10-19 | 0.00 | 0.01 | 0.02 | 0.00 | 0.02 | 0.01 | 0.00 | 0.01 | 0.00 | 0.00 |  |
|  | 20-29 | 0.00 | 0.00 | 0.05 | 0.00 | 0.03 | 0.03 | 0.01 | 0.02 | 0.01 | 0.00 |  |
|  | 30-39 | 0.00 | 0.00 | 0.01 | 0.05 | 0.03 | 0.02 | 0.02 | 0.02 | 0.01 | 0.00 |  |
|  | 40-49 | 0.00 | 0.00 | 0.01 | 0.02 | 0.07 | 0.05 | 0.00 | 0.01 | 0.02 | 0.00 |  |
|  | 50-59 | 0.00 | 0.00 | 0.04 | 0.01 | 0.05 | 0.06 | 0.01 | 0.04 | 0.03 | 0.01 |  |
|  | 60-69 | 0.00 | 0.00 | 0.01 | 0.00 | 0.01 | 0.01 | 0.02 | 0.02 | 0.02 | 0.00 |  |
|  | 70-79 | 0.00 | 0.00 | 0.01 | 0.00 | 0.02 | 0.00 | 0.01 | 0.02 | 0.03 | 0.00 |  |
|  | 80-89 | 0.00 | 0.00 | 0.00 | 0.00 | 0.00 | 0.01 | 0.00 | 0.00 | 0.01 | 0.00 |  |
|  | ≥90 | 0.00 | 0.00 | 0.00 | 0.00 | 0.00 | 0.00 | 0.00 | 0.00 | 0.01 | 0.00 |  |

|  |  |  |  |  |  |  |  |  |  |  |  |
| --- | --- | --- | --- | --- | --- | --- | --- | --- | --- | --- | --- |

**Table S4. Observed age-specific transmission probability matrix in the second epidemic wave in South Korea.** The probability is derived as the density measure by using the respective frequencies for the transmission among the age-groups of infector-infectee.

|  | | Infectee | | | | | | | | | | |
| --- | --- | --- | --- | --- | --- | --- | --- | --- | --- | --- | --- | --- |
|  |  | 0-9 | 10-19 | 20-29 | 30-39 | 40-49 | 50-59 | 60-69 | 70-79 | 80-89 | ≥90 |  |
| Infector | 0-9 | 0.00 | 0.00 | 0.00 | 0.00 | 0.00 | 0.00 | 0.00 | 0.00 | 0.00 | 0.00 |  |
|  | 10-19 | 0.00 | 0.00 | 0.00 | 0.00 | 0.01 | 0.00 | 0.00 | 0.00 | 0.00 | 0.00 |  |
|  | 20-29 | 0.01 | 0.00 | 0.06 | 0.03 | 0.02 | 0.03 | 0.03 | 0.00 | 0.00 | 0.00 |  |
|  | 30-39 | 0.02 | 0.01 | 0.02 | 0.05 | 0.02 | 0.01 | 0.02 | 0.00 | 0.00 | 0.00 |  |
|  | 40-49 | 0.00 | 0.00 | 0.02 | 0.01 | 0.05 | 0.03 | 0.03 | 0.00 | 0.00 | 0.00 |  |
|  | 50-59 | 0.00 | 0.00 | 0.02 | 0.02 | 0.03 | 0.02 | 0.04 | 0.01 | 0.00 | 0.00 |  |
|  | 60-69 | 0.01 | 0.00 | 0.01 | 0.04 | 0.04 | 0.03 | 0.07 | 0.03 | 0.01 | 0.00 |  |
|  | 70-79 | 0.00 | 0.00 | 0.00 | 0.01 | 0.02 | 0.02 | 0.02 | 0.02 | 0.01 | 0.00 |  |
|  | 80-89 | 0.00 | 0.00 | 0.00 | 0.00 | 0.00 | 0.00 | 0.00 | 0.00 | 0.00 | 0.00 |  |
|  | ≥90 | 0.00 | 0.00 | 0.00 | 0.00 | 0.00 | 0.00 | 0.00 | 0.00 | 0.00 | 0.00 |  |

**Table S5. Estimated age-specific proportions of asymptomatic SARS-CoV-2 infection at presentation among all local cases**

|  | First epidemic wave | | | | Second epidemic wave | | | |
| --- | --- | --- | --- | --- | --- | --- | --- | --- |
| Age group (years) | No. of asymptomatic cases | No. of confirmed cases | NA | Proportion of asymptomatic (95% CI^†^), % | No. of asymptomatic cases | No. of confirmed cases | NA | Proportion of asymptomatic (95% CI^†^), % |
| 0–19 | 23 | 100 | 23 | 29.9 (20.8–40.8) | 42 | 150 | 44 | 39.6 (30.8–49.1) |
| 20–39 | 49 | 475 | 142 | 14.7 (11.3– 18.9) | 83 | 539 | 159 | 21.8 (18.0–26.3) |
| 40–59 | 107 | 576 | 158 | 25.6 (21.6–30.0) | 92 | 551 | 177 | 24.6 (20.5–29.2) |
| 60–79 | 44 | 261 | 75 | 23.7 (18.1–30.3) | 130 | 570 | 159 | 31.6 (27.3–36.3) |
| ≥80 | 11 | 49 | 8 | 26.8 (15.7–41.9) | 32 | 93 | 35 | 55.2 (42.5–67.3) |
| NA | 0 | 22 | 19 | -^‡^ | 21 | 427 | 281 | 14.4 (9.6-21.0) |
| Overall | 234 | 1483 | 406 | 21.7 (19.4–24.3) | 400 | 2330 | 855 | 27.1 (24.9–29.4) |

†CI, Confidence interval

^‡^ The estimate was not available due to the very low sample size

NA: Not available

Note. The first epidemic period is from 19 January to 19 April 2020, and the second epidemic period is from the 20 April to 11 August 2020. The 95% confidence interval was estimated by the binomial method.

**Table S6. Estimated age-specific proportions of unlinked COVID-19 cases in South Korea.**

|  | First epidemic wave | | | Second epidemic wave | | |
| --- | --- | --- | --- | --- | --- | --- |
| Age group (years) | No. of unlinked cases | No. of confirmed cases | Proportion of unlinked (95% CI^†^), % | No. of unlinked cases | No. of confirmed cases | Proportion of unlinked (95% CI^†^), % |
| 0–19 | 15 | 100 | 15.0 (9.3–23.3) | 26 | 150 | 17.3 (12.1–24.2) |
| 20–39 | 124 | 475 | 26.1 (22.4–30.2) | 117 | 539 | 21.7 (18.4–25.4) |
| 40–59 | 113 | 576 | 19.6 (16.6–23.1) | 133 | 551 | 24.1 (20.8–27.9) |
| 60–79 | 60 | 261 | 23.0 (18.3–28.5) | 136 | 570 | 23.9 (20.5–27.5) |
| ≥80 | 10 | 49 | 20.4 (11.5–33.6) | 16 | 93 | 17.2 (10.9–26.1) |
| NA | 4 | 22 | 18.2 (7.3-38.5) | 162 | 427 | 37.9 (33.5-42.6) |
| Overall | 326 | 1483 | 22.0 (19.9–24.2) | 432 | 2330 | 22.5 (20.7–24.4) |

†CI, Confidence interval

Note. The first wave is from 19 January to 19 April 2020, and the second wave is from the 20 April to 11 August 2020. The 95% confidence interval was estimated by the binomial method.
